# The scalable variant call representation: enabling genetic analysis beyond one million genomes

**DOI:** 10.1093/bioinformatics/btae746

**Published:** 2024-12-24

**Authors:** Timothy Poterba, Christopher Vittal, Daniel King, Daniel Goldstein, Jacqueline I Goldstein, Patrick Schultz, Konrad J Karczewski, Cotton Seed, Benjamin M Neale

**Affiliations:** Program in Medical and Population Genetics, Broad Institute of MIT and Harvard, Cambridge, MA 02142, United States; Analytic and Translational Genetics Unit, Massachusetts General Hospital, Boston, MA 02114, United States; Stanley Center for Psychiatric Research, Broad Institute of MIT and Harvard, Cambridge, MA 02142, United States; Program in Medical and Population Genetics, Broad Institute of MIT and Harvard, Cambridge, MA 02142, United States; Analytic and Translational Genetics Unit, Massachusetts General Hospital, Boston, MA 02114, United States; Stanley Center for Psychiatric Research, Broad Institute of MIT and Harvard, Cambridge, MA 02142, United States; Program in Medical and Population Genetics, Broad Institute of MIT and Harvard, Cambridge, MA 02142, United States; Analytic and Translational Genetics Unit, Massachusetts General Hospital, Boston, MA 02114, United States; Stanley Center for Psychiatric Research, Broad Institute of MIT and Harvard, Cambridge, MA 02142, United States; Novo Nordisk Foundation Center for Genomic Mechanisms of Disease, Broad Institute of MIT and Harvard, Cambridge, MA 02142, United States; Program in Medical and Population Genetics, Broad Institute of MIT and Harvard, Cambridge, MA 02142, United States; Analytic and Translational Genetics Unit, Massachusetts General Hospital, Boston, MA 02114, United States; Stanley Center for Psychiatric Research, Broad Institute of MIT and Harvard, Cambridge, MA 02142, United States; Program in Medical and Population Genetics, Broad Institute of MIT and Harvard, Cambridge, MA 02142, United States; Analytic and Translational Genetics Unit, Massachusetts General Hospital, Boston, MA 02114, United States; Stanley Center for Psychiatric Research, Broad Institute of MIT and Harvard, Cambridge, MA 02142, United States; Program in Medical and Population Genetics, Broad Institute of MIT and Harvard, Cambridge, MA 02142, United States; Analytic and Translational Genetics Unit, Massachusetts General Hospital, Boston, MA 02114, United States; Stanley Center for Psychiatric Research, Broad Institute of MIT and Harvard, Cambridge, MA 02142, United States; Program in Medical and Population Genetics, Broad Institute of MIT and Harvard, Cambridge, MA 02142, United States; Analytic and Translational Genetics Unit, Massachusetts General Hospital, Boston, MA 02114, United States; Stanley Center for Psychiatric Research, Broad Institute of MIT and Harvard, Cambridge, MA 02142, United States; Novo Nordisk Foundation Center for Genomic Mechanisms of Disease, Broad Institute of MIT and Harvard, Cambridge, MA 02142, United States; Program in Medical and Population Genetics, Broad Institute of MIT and Harvard, Cambridge, MA 02142, United States; Analytic and Translational Genetics Unit, Massachusetts General Hospital, Boston, MA 02114, United States; Stanley Center for Psychiatric Research, Broad Institute of MIT and Harvard, Cambridge, MA 02142, United States; Novo Nordisk Foundation Center for Genomic Mechanisms of Disease, Broad Institute of MIT and Harvard, Cambridge, MA 02142, United States; Program in Medical and Population Genetics, Broad Institute of MIT and Harvard, Cambridge, MA 02142, United States; Analytic and Translational Genetics Unit, Massachusetts General Hospital, Boston, MA 02114, United States; Stanley Center for Psychiatric Research, Broad Institute of MIT and Harvard, Cambridge, MA 02142, United States; Novo Nordisk Foundation Center for Genomic Mechanisms of Disease, Broad Institute of MIT and Harvard, Cambridge, MA 02142, United States

## Abstract

**Motivation:**

The Variant Call Format (VCF) is widely used in genome sequencing but scales poorly. For instance, we estimate a 150 000 genome VCF would occupy 900 TiB, making it costly and complicated to produce, analyze, and store. The issue stems from VCF’s requirement to densely represent both reference-genotypes and allele-indexed arrays. These requirements lead to unnecessary data duplication and, ultimately, very large files.

**Results:**

To address these challenges, we introduce the Scalable Variant Call Representation (SVCR). This representation reduces file sizes by ensuring they scale linearly with samples. SVCR’s linear scaling relies on two techniques, both necessary for linearity: local allele indices and reference blocks, which were first introduced by the Genomic Variant Call Format. SVCR is also lossless and mergeable, allowing for *N* + 1 and *N* + *K* incremental joint-calling. We present two implementations of SVCR: SVCR-VCF, which encodes SVCR in VCF format, and VDS, which uses Hail’s native format. Our experiments confirm the linear scalability of SVCR-VCF and VDS, in contrast to the super-linear growth seen with standard VCF files. We also discuss the VDS Combiner, a scalable, open-source tool for producing a VDS from GVCFs and unique features of VDS which enable rapid data analysis. SVCR, and VDS in particular, ensure the scientific community can generate, analyze, and disseminate genetics datasets with millions of samples.

**Availability and implementation:**

https://github.com/hail-is/hail/.

## 1 Introduction

The pipeline for high-throughput sequencing involves a series of datatypes and the transformations between them:

1. Sequencing DNA to generate unaligned reads, often stored in a FASTQ.
2. Aligning to a reference genome to generate aligned reads, often stored in a BAM/CRAM.
3. Variant calling to generate genotype calls and metadata, often stored in a Genomic Variant Call Format (GVCF) or Variant Call Format (VCF).
4. Joint calling to generate QC/analysis-ready sequence-matrix, often stored in a VCF.

Sequencing, aligning, and variant calling are straightforwardly sample-parallel, but joint calling is not. The latter necessarily combines multiple sample-oriented files into a single variant-oriented matrix. In this paper, we focus exclusively on joint calling and its data formats. Moreover, we only consider data formats which support sequencing quality information, such as per-allele read depths and per-genotype likelihoods, rather than restricting to hardcalls (e.g. Plink). These formats are critical as joint calling is always followed by cohort-level sequencing quality control before performing downstream analyses such as GWAS.

When cohort sizes were small, variant calling and joint calling were often performed within a single process (e.g. using GATK UnifiedGenotyper). As cohorts grew, these steps were decoupled by introducing an intermediate file format, GVCF, a single-sample VCF in which intervals containing only homozygous reference calls are sparsely stored (e.g. created through GATK HaplotypeCaller). Moreover, the term Project VCF (PVCF) was introduced to describe the convention of representing multiple samples in a VCF without variant sites containing only homozygous reference calls. This decoupling allowed sample-parallel variant calling to scale up, but did not address the scaling challenges of joint calling.

In the intervening years, cohorts continued to grow until combining many GVCFs into one PVCF became challenging. In response, the community developed new tools (e.g. DRAGEN gVCF Genotyper, GLNexus), new representations (e.g. msVCF, spvcf), new compressors (e.g. popvcf), and new formats (e.g. Savvy, Genomic Variant Store, GLNexus’s key-value store, GenomicsDB). These efforts draw from three approaches: sparsity of variant genotypes, sparsity of alleles, and amenability to sample parallelism. In this paper, we describe a framework which employs all three techniques to address the above challenges and yield exactly linear scaling of size in samples, sample parallelism in generation, incremental *N* + *K* mergeability, and a plaintext VCF representation.

Formats and representations: The common representation of genomic sequences, Project VCF (PVCF), is untenable at scale. To see why, consider the addition of a single sample to a sequencing dataset with N samples. PVCF requires, for every novel variant in the new sample, an additional row containing one variant genotype and N homozygous reference genotypes. Furthermore, each new allele that the *N* + 1th sample introduces requires extension of existing fields to account for the new allele. For instance, the first N samples’ allele depth (AD) fields need a new “0” (no reads) entry for the new allele. Worse, the Phred-scaled genotype likelihood (PL) field grows quadratically in the number of alleles. The result is a repetitive and bloated representation, where the same uninformative value is stored over and over again, leading to inefficient use of storage and computational resources.

Throughout this paper we distinguish between a representation and a format. A representation, or data model, defines the expected set of fields, what they mean, and how they are related. A format describes a concrete implementation in terms of bytes. We similarly cleave the VCF Specification into the PVCF representation for cohort-level variant data and the VCF format for storing a variant-by-sample matrix of arbitrary data types in tab-delimited plaintext.

Specifically, PVCF (as a representation) defines the semantics of fields such as GT, AD, GP, PL, and, for list fields, the relationship between their length and the number of alternate alleles. VCF (as a format) describes, e.g. how a number or a list is rendered in plaintext. The VCF supports representations other than PVCF: the single-sample GVCF and the structural variant VCF (SV VCF). Analogously, the PVCF representation supports different formats: VCF and Binary Call Format (BCF).

Variant sites scale with samples. When discussing the size of a dataset, we focus on the number of genotype records. The number of genotype records in a GVCF does not vary substantially across samples. We use K to refer to the average number of nonreference genotype records per sample, which, at 3–6 million, is much smaller than the number of bases in the human genome (3 billion).

In a multi-sample dataset, the expected number of loci at which at least one sample has a nonreference genotype is a function of the number of samples, *M*(*N*). We call such loci variant sites, reserving the term variant to mean a particular allele at a particular locus. We use the term genotype record to refer to a genotype call and its associated quality metrics. By definition, *M*(1) = *K* and *M*(*N*), for large *N*, approaches the number of loci in the reference genome. In contrast, the number of variants is unbounded due to the infinitude of possible insertion and deletion variants.

Consider *M*(2): The second sample will share many variant sites with the first sample; however, both samples will have many unshared singletons. As a result, this second sample substantially increases the number of variant sites in the dataset. In contrast, the millionth sample will add significantly fewer variant sites because the previous 999 999 samples are likely to share many of its sites. Thus, *M*(*N*) is nonlinear, which we confirm below.

PVCF represents a collection of sequences as a dense matrix, with one column per sequenced sample and one row for every variant site. PVCF permits both a multiallelic representation (wherein each locus appears in at most one row) and a biallelic representation (wherein loci are repeated such that each row contains one alternate allele). The number of genotype records in a multiallelic PVCF grows as

O(N×M(N))

and the number of genotype records in a biallelic PVCF grows as

O(N×M(N)×A(N))

where *A*(*N*) is the expected number of alternate alleles at a variant site in a dataset with *N* samples.

Until variants are observed at all loci, each sample brings with it some number of new singleton sites. We empirically measured the relationship between the number of variant sites and the number of samples in subsets of the PVCF representation of the HGDP + 1kG dataset. We found that the number of variant sites grows roughly with the square-root of N. We measure the exponential relationship between M and N using a linear model in log-space:

$$\begin{array}{c} \;{\text{log}}_{10}\left(M\right)=a+{b\;\text{log}\;}_{10}\left(N\right)\\ M=10^{a}N^{b} \end{array}$$

**Figure 1.**
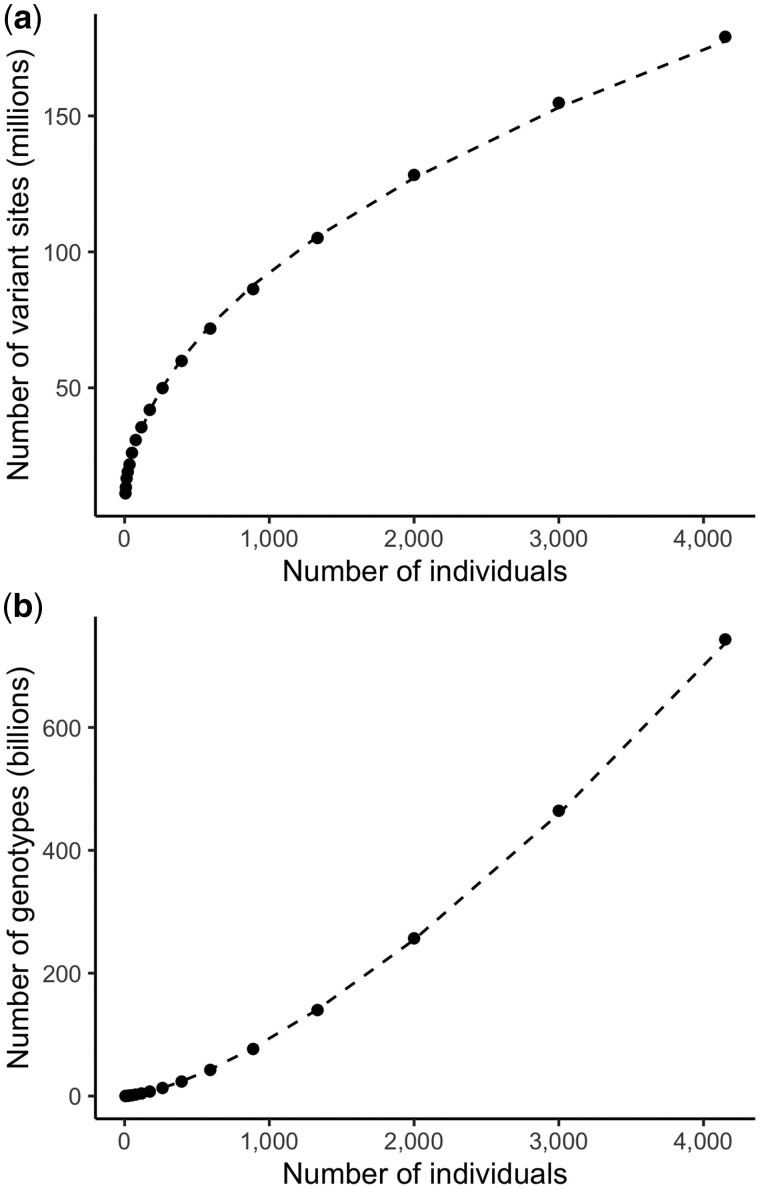
The number of genotype records and variant sites in subsets of the HGDP + 1kG dataset. The number of variant sites (a) and total nonreference genotypes (b) scale with the number of samples. Each new sample brings new variant sites (a) which causes the dense matrix to grow super-linearly (b) in the number of samples. Dashed line indicates linear fit in log-log space, using all points with at least 100 individuals, with fit lines (a) 0.457*log_10_(variants) + 6.59, and (b) 1.457*log_10_(genotypes) + 6.59.

To explore this relationship, we generated multiallelic PVCFs from subsets of the HGDP + 1kG dataset (Koenig *et al.* 2023). Figure 1a shows, for HGDP + 1kG, *M* ≈ *N*^0.46^. Figure 1b shows the effect of this growth on the number of genotypes. The same experiment applied to subsets of gnomAD v2 found *M* ≈ *N*^0.57^. We stress that this is only evidence of an exponential relationship when *N* is between one and several thousand. Furthermore, this relationship depends on the ancestry mix of the dataset: diverse ancestries will increase the rate at which new variant sites are discovered. As the number of samples grows, the growth of variant sites will eventually decelerate, although we are yet to reach this regime.

Fields scale with alleles: In multiallelic PVCFs, not only does the number of genotype records grow super-linearly, but the size of each genotype record increases with the size of the cohort. In particular, sequencing datasets store per-allele quality metadata. For example, each genotype record stores an allele depth (AD) field containing one integer per allele observed at this locus. This field grows linearly in the number of alleles at the locus even though most genotypes observe at most two alleles. Even worse, the phred-scaled genotype likelihood (PL) field stores the likelihood of all possible genotypes given the alleles at this locus. Within the autosomes, which are diploid, this field grows quadratically in the number of alleles. In practice, particularly in repetitive regions (e.g. telomeres), a small fraction of loci have thousands to tens of thousands of alleles.

In summary, if *A*(*N*) is the average number of alleles per site, then the total number of allele depths in a multiallelic PVCF file grows as,

O(N×M(N)×A(N))

and the total number of phred-scaled genotype likelihoods grows as,

$$O\left(N\times M\left(N\right)\times A{\left(N\right)}^{2}\right)$$

In practice, at most sites we observe 2–4 alternate alleles, but at a small number of sites, we observe thousands of alternate alleles. Sites like these are particularly challenging to analyze because of the quadratic growth of the PL field. We conjecture this quadratic growth explains why many PVCFs with more than a few thousand samples have dropped the PL field entirely or dropped it from multiallelic loci.

Notice that both multiallelic and biallelic datasets suffer from two forms of nonlinear growth (genotypes and alleles). They differ only in where the growth appears. In the multiallelic representation, both genotype records and allele indexed fields grow super-linearly in the number of samples. In the biallelic representation, allele-indexed fields are bounded to length three but the number of genotype records grows even faster because highly multiallelic variant sites duplicate reference calls across every allele.

## 2 Materials and methods

We propose a new representation for sequenced cohorts: the Scalable Variant Call Representation or SVCR. This representation is a generalization of GVCF to more than one sample. SVCR scales linearly in the number of samples. One or more GVCFs can be losslessly converted to a single SVCR dataset. Two SVCR datasets can be losslessly merged into a new SVCR dataset comprising samples from both. An SVCR dataset can be stored in many formats including VCF, Hail native format, and Google BigQuery.

### 2.1 Overview

A dataset in the SVCR representation, which has a set of samples, consists of zero or more variant sites. Variant sites are indexed and identified by genomic locus: thus, there is at most one variant site per locus, i.e. variant sites can be multi-allelic. Every variant site has a genotype record for every sample. A genotype record is either the unique missing value or a set of fields and their values. Any field value may be missing. Any value permitted for a VCF FORMAT field is permitted as a field value in SVCR.

### 2.2 Column-sparsity

Within a single sample, SVCR stores adjacent reference genotype records as intervals, which are termed “reference blocks.” Each reference block has a locus interval, which must be contained within a single chromosome. A reference block is stored in the variant site identified by the interval’s first, or left-hand, locus. The size of the reference block, in base pairs, is stored in a genotype field named *LEN*. All reference block intervals for any particular sample must be disjoint.

Reference blocks are a form of column-sparsity by run-length encoding. At each locus, the genotype record of samples with a homozygous reference genotype is encoded in the underlying format as a missing value. The actual value is implicitly given by that sample’s overlapping reference block. Preservation of the reference blocks enables the combination of two or more SVCR datasets into a new SVCR dataset.

### 2.3 Local alleles

Consider a highly polymorphic low-complexity insertion-deletion locus with 1000 alternate alleles in a PVCF with 100 000 whole genomes. The VCF spec mandates that the allelic depth field *AD* has one value per allele including the reference (*R-numbered*), so AD records at this site must contain 1000 elements denoting the depth of reads supporting each allele. For any particular sample, it is likely that at most two of these alternate alleles are observed. In the PVCF representation, the *AD* field for such a sample would contain between 998 and 999 zeros. The GVCF representation, in contrast, contains at most three entries. As described previously, this problem is significantly worse for quadratic fields like Phred-scaled genotype likelihoods (*PL*), which have one value for each possible genotype configuration (*G-numbered*). Data generators have mitigated this problem with approaches such as truncating the list of alternate alleles at any given locus to a maximum number, or disseminating PVCFs with only genotype (*GT*) fields and no quality metadata. Each of these approaches is necessarily lossy and has drawbacks: truncating alternate alleles removes potentially pathogenic information from the dataset and masks information from short tandem repeats (STRs), and dropping all quality fields makes quality control much more difficult.

**Figure 2.**
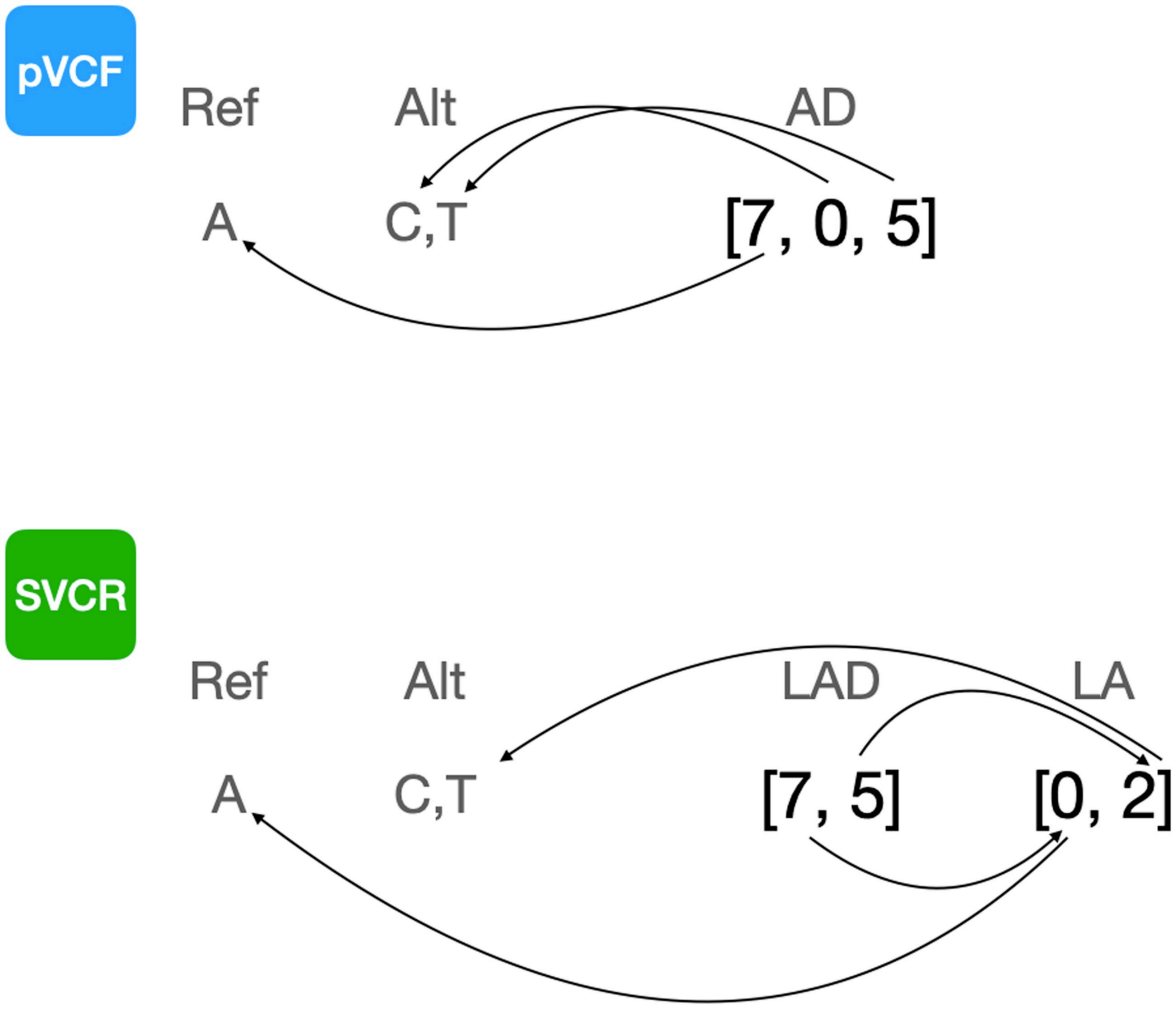
Local alleles. The AD field is an array field containing one element per allele, including the reference. The AD field entries indicate, for each allele, the number of informative reads. The arrows terminating at an allele indicate to which allele each value corresponds. The LAD field is a locally indexed array field storing the same data. Note that we need not store information for the unobserved C allele, and that the two-step paths lead to the same alleles as the one-step path.

SVCR introduces a new entry-level field, *local alleles (LA)*, a topic introduced in the hts-specs library through the discussion of this work (https://github.com/samtools/hts-specs/pull/434). At each locus, for each sample, an LA field indicates which alleles were observed in this sample. The *global alleles* refers to the set of alleles observed in any sample at this locus. Concretely, the LA field is an injective function from local allele indices to global allele indices (Fig. 2). Since the number of local allele indices is finite (in a diploid genotype, there are at most three), we represent the LA field as an array. For example, at a locus with 1000 global alleles, an LA value of [0, 4, 5] indicates that the first local allele corresponds to the first (or reference) global allele, the second local allele corresponds to the fifth global allele, and the third local allele corresponds to the sixth global allele. For convenience, we require the first element of LA to always be zero.

For each genotype-level allele-indexed field from the VCF spec, we define a new field whose name begins with “L.” The field only contains the elements corresponding to this sample’s local alleles. Consider a variant site with five alleles, A, AA, AAA, AAAA, AAAAA, and a sample with the genotype AA/AAAA with 14 reads supporting AA and 16 reads supporting AAAA. The local alleles array will be [0, 1, 3], the allelic depth (AD) will be [0, 14, 0, 16, 0], and the local allelic depth (LAD) array will be [0, 14, 16].

Although the GT field is not problematic, we recommend using an LGT field in SVCR. In particular, when removing a sample, an implementation of SVCR may wish to remove global alleles which no longer appear in any samples. Removing a global allele requires modifying every local alleles entry *and also* updating any globally indexed fields, such as the GT.

### 2.4 SVCR-VCF

We encode SVCR in VCF by requiring two FORMAT fields: LA, an array of integers, and LEN, an integer (described above). Reference blocks must have a nonmissing LEN, should have a nonmissing DP and GQ, may have a nonmissing LGT, and should have a missing LA, LAD, and LPL. For a given sample, at every locus between a reference block’s first locus and its last locus, every FORMAT field, for the given sample, must be missing. Nonreference genotypes must have a missing LEN, must have a nonmissing LA, and should have a nonmissing DP, GQ, LGT, LAD, and LPL. For convenience, we recommend storing the length of the longest reference block in a meta-information (header) line with the key MAXIMUM_REFERENCE_BLOCK_LENGTH.

We explicitly define the new fields as follows (but encourage users to apply the local allele indexing technique to every A-, R-, and G-numbered VCF field):

- LA, the local alleles. An injective map, represented as an array, from local allele indices (e.g. 0, 1, 2) to global allele indices. The reference allele has global allele index 0. The global allele index of each alternate allele is one more than its index in ALT.
- LGT, the local genotype. Defined exactly as GT is by the VCF 4.4 specification except that the “allele values” are instead indices (e.g. 0, 1, 2) into the LA field.
- LAD, the local allele depth. The per-sample read depths for each local allele. See the previous section and Fig. 2 for examples.
- LPL, the local Phred-scaled genotype likelihoods. Defined exactly as PL is by the VCF 4.4 specification except we only include entries for all possible genotypes given the set of local alleles.
- SVCR introduces three new settings for the “Number” of a VCF list field:
- LA-numbered fields should have one list element for each allele present in LA minus one (to exclude the reference).
- LR-numbered fields should have one list element for each allele present in LA (including the reference). For example: LAD.
- LG-numbered fields should have one list element for each possible genotype depending on the number of alleles NLA (including the reference allele) and the ploidy of the genotype call. If diploid, (NLA * (NLA+1))/2. If haploid, NLA. For example: LPL.

**Figure 3.**
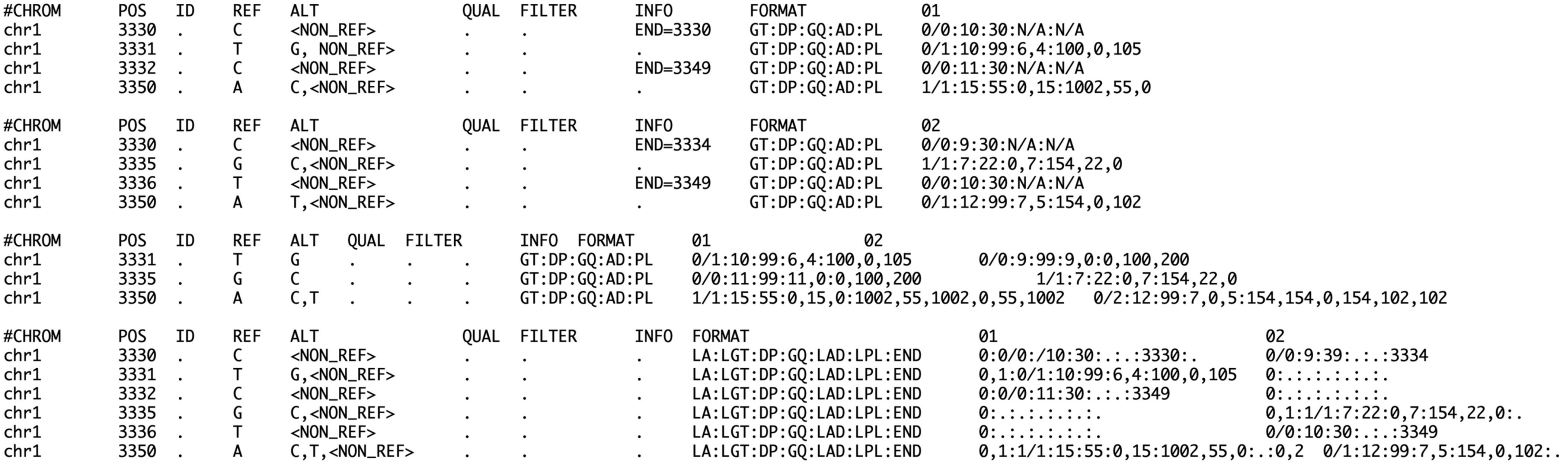
Representing sequences. In each panel, two different samples, 01 and 02, are shown in different representations, including two GVCF files (top two), a PVCF file (third), and an SVCR-VCF file (last).

Figure 3 demonstrates the equivalent representation of twenty base pairs of two samples in GVCF, PVCF, and SVCR-VCF.

**Figure 4.**
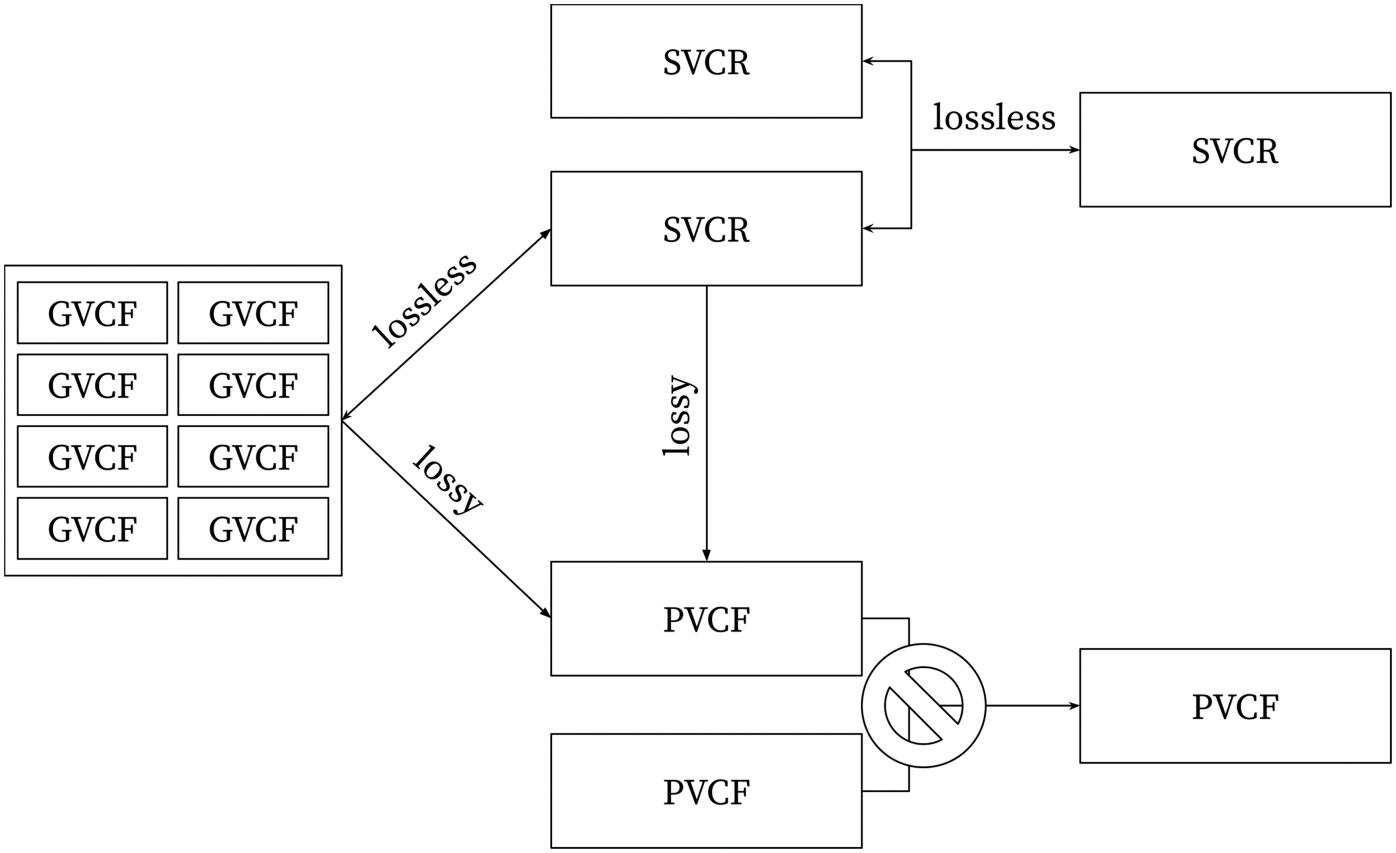
The graph of interconversion and combination operations. Notice that PVCF is a sink: crucial information is lost when producing a PVCF—missing data and homozygous reference calls cannot be unambiguously resolved if the quality metrics at nonvariant sites are discarded, and thus, these formats cannot be reliably combined.

### 2.5 SVCR-VCF and extant tools

During the VCF format’s 13 year life (Danecek *et al.* 2011), the community has built many tools, libraries, and scripts. Like GVCF and Structural Variant VCF, SVCR-VCF files defy some of the expectations of these extant tools, libraries, and scripts. For example, software that is unaware of reference blocks will incorrectly interpret homozygous reference calls in SVCR-VCF (other than the beginning of the reference block) as missing, which may impact, e.g. allele frequency calculation. Moreover, software that expects a GT field, e.g. a frequency-based relatedness estimator, will either error, crash, or treat every genotype as missing.

We make two recommendations to the community:

1. We agree with the draft VCF 4.5 specification which recommends “that VCF libraries provide an API in which […] values [are] accessed through their corresponding nonlocal key.” We also support with the implicit recommendation to replace bespoke and partly compliant VCF parsers with a small number of compliant and fast parsers which abstract the details of the format and representation.
2. Due to the popularity of tools that only consult the GT field, we recommend data producers share both an SVCR-VCF and an already-quality-controlled “hardcalls” PVCF. A “hardcalls” PVCF is one which includes no A-, R-, and G-numbered FORMAT fields and does not contain reference blocks. It is simply a dense matrix of high quality genotypes.

In the related work section, we discuss tools that have already adopted local allele indexing or reference blocks.

VDS: We also encode SVCR in a Hail native format called Variant Dataset or VDS (unrelated to the Hail 0.1 VariantDataset except in name). A VDS comprises two Hail matrix tables. One Hail matrix table contains reference data the other contains variant data. The reference matrix table has rows keyed by locus, columns keyed by sample identifier, and three entry fields: LEN (int32), DP (int32), and GQ (int32). Entries which are not the start of a reference block are missing values.

The variant matrix table has rows keyed by locus and alleles, columns keyed by sample id, and at least two entry fields, LA (array<int32>) and LGT (call), and usually also has LAD (array<int32>), LPL (array<int32>), DP (int32), GQ (int32), and gvcf_info (struct<…>). The latter contains all the GVCF INFO fields for that sample.

For data interchange, we recommend a format that uses a single file. For analysis, we recommend storing reference and variant data in two files for three reasons:

1. The schemas for reference and variant records differ; therefore, a combined representation pays the overhead of mixing these schemas. We observed a roughly 10% size reduction in both VCF and VDS from a split format.
2. The split representation makes it easier to filter variants without inadvertently removing reference blocks.
3. Analytical and quality control methods that operate on the smaller variant-only table are faster and cheaper because they read substantially less data. For example, the newly developed CHARR (Lu *et al.* 2023) achieves substantial speedups as it only needs to access variant data (homozygous genotypes).

### 2.6 Sparsity in representation versus sparsity in format

It should be noted that while SVCR is sparse—only a small number of matrix entries contain information—both the GZIP-compressed VCF and Hail VDS realizations of SVCR contain high numbers of explicit “missing” values. Both formats use compression techniques which rapidly and efficiently compress and decompress these repeated values, reducing the gain from switching to an explicitly sparse format. We anticipate substantial additional reductions in analysis cost and time from a system that stores and computes directly on the sparse representation.

### 2.7 Densification

The reference information for all samples at some locus L is not contained within a single row of the matrix at L. Instead, this information is stored in overlapping reference blocks that appear at their start location, which is necessarily some previous locus. We call producing a single row containing all reference and variant information for all samples at a particular locus densification. In general, densifying a locus requires searching all previous loci until an overlapping reference block for every sample has been identified.

### 2.8 Mitigation by periodic reference checkpoints

We propose two strategies to bound the size of each reference block and thus bound the search distance. Reference blocks could either be split at some fixed size limit or at fixed intervals over the genome. Both strategies add reference blocks and therefore trade increased space for reduced densification time. Both strategies have a parameter K such that, at a particular variant site, for all samples, all overlapping reference blocks begin no further than K base pairs prior.

Splitting at a fixed period: Split reference blocks at every *K* bases. Store reference blocks for all nonvariant samples at each split location. If this dataset is also partitioned into *K*-sized partitions, the information necessary to realize any partition’s dense sub-matrix of genotypes is fully contained in that partition. This approach inflates the reference data by splitting every reference block that spans the *K*th locus even if that reference block is already tiny. As shown below, this inflation relative to the next strategy is very modest.

Splitting at a size threshold: Split reference blocks spanning more than *K* bases into two or more reference blocks spanning at most K bases. For instance, with a value of *K* = 10 000 a reference block starting at chr1:4500 with length 38 000 would be split into reference blocks chr1:4500–14499, chr1:14500–24499, chr1:24500–34499, chr1:34500–42499. This strategy introduces the minimum number of new reference blocks to ensure the K invariant; however, in partitioned datasets, data from previous partitions may be necessary to realize the dense sub-matrix of genotypes at any partition after the first.

*K* is a parameter of the dataset: We do not mandate or recommend any particular value of *K* because the length distribution of reference blocks substantially depends on experimental design, variant calling, and reference block bin resolution. For example, whole exome sequencing produces shorter reference blocks due to the fluctuations in depth from partial capture of the genome. In contrast, high coverage whole genome sequencing can produce long blocks of similar-quality reference calls in regions of constant depth. Tables 1 and 2 present the measured number of reference blocks after splitting the HGDP + 1kG dataset using each strategy.

**Table 1.** Data size is inversely proportional to the period when splitting at a fixed period.^a^

K (fixed period)	Billions of reference blocks after splitting	Ratio of split to unsplit reference blocks
50 000	73.1	1.0000
10 000	73.1	1.0002
5000	73.2	1.0015
1000	77.7	1.0634
500	87.1	1.1925
100	175.5	2.4013

a Using this strategy, a period of 5000 bases introduces a modest 0.15% increase in size. The increased sizes of other periods are also shown. At a period of 50 000 bases, only a few hundred reference blocks are added.

**Table 2.** Data size is inversely proportional to the threshold when splitting at a size threshold.^a^

K (size threshold)	Billions of reference blocks after splitting	Ratio of split to unsplit reference blocks
50 000	73.1	1.0000
10 000	73.1	1.0002
5000	73.2	1.0015
1000	77.7	1.0634
500	87.1	1.1922
100	175.3	2.3985

a In practice, compared to splitting at a fixed period, this strategy is only mildly more parsimonious.

In our experience, the granularity of reference block GQ bins has a large impact on the average length of reference blocks in a given dataset. Whole genome sequencing data also tends to have longer reference blocks than whole exome data, due to the absence of fixed capture boundaries and more consistent coverage across the genome.

### 2.9 Streaming densification

The previous discussion and mitigations address reading one particular locus, which we call a “point-query.” In contrast, applications that read all or most of the dataset do not need these mitigations. Instead, streaming densification keeps one overlapping reference block per sample, swapping in new reference blocks as they appear in the stream of records. As stated above, this dense matrix is super-linear in size. Although a streaming application does not reify the entire matrix in memory, if it performs computation on each dense entry, the compute-time, and thus cost, will grow super-linearly. For example, computing the allele frequency necessarily inspects averages over every call, including the homozygous reference calls. A future implementation that avoids densification will increase performance for these queries.

### 2.10 GVCF is a single-sample SVCR

SVCR is a multi-sample generalization of GVCF. Indeed, an SVCR with one sample is equivalent to a GVCFS (Fig. 4). In a GVCF, the global alleles and the local alleles are the same because there is only one sample. In this setting, locally indexed and globally indexed fields are equivalent. Specifically, in a GVCF, the *AD* and *PL* fields must be equal to the *LAD* and *LPL* fields. The lossless transformation between a GVCF and SVCR-VCF is as simple as (i) adding a field LA to create an identity function of the alleles, (ii) adding an “L” prefix to the name of allele-indexed fields, (iii) adding a *LEN* field to the FORMAT derived from the position and the *END* field.

### 2.11 Hierarchical joint calling

An SVCR may be produced in a tree-structured manner because it is combinable with itself and with GVCFs. This allows some sample-wise parallelism in the production of an SVCR dataset from GVCFs. Suppose we have 100 000 GVCFs. We may, in parallel, combine 1000 groups of 100 GVCFs each into 1000 SVCR datasets. We may then, in parallel, combine ten groups of 100 SVCR datasets into ten SVCR datasets. Finally, we may combine the ten SVCR datasets into one SVCR dataset. This entire process is also fully variant parallel: in particular, note that reference blocks from GVCFs or smaller SVCRs are simply preserved at their locus and have no impact on any other locus.

## 3 Results

### 3.1 Joint calling and analysis of nearly one million exomes

VDS, the SVCR implementation in Hail format, has been used since 2019 to merge, store, and analyze large sequencing cohorts. We note that although much of these results here are based on VDS, the essential property for these successful projects was not the particulars of the VDS format but rather the fact that the SVCR representation grows linearly in the number of samples. Without this fact, the storage costs alone, already a significant cost burden, would be 300–1000 times as high.

The Hail library includes a “VDS Combiner” that can combine zero or more VDSes with zero or more GVCFs into a single VDS in a parallel and fault-tolerant manner. Five large datasets produced by the VDS Combiner directly from GVCF files are:

1. gnomAD v3.1. 153 000 genomes. 2019. (Chen *et al.* 2024).
2. Center for Common Disease Genomics (CCDG) exomes. 203 000 exomes. 2021. (https://www.genome.gov/Funded-Programs-Projects/NHGRI-Genome-Sequencing-Program/Centers-for-Common-Disease-Genomics).
3. CCDG genomes. 136 000 genomes. 2021.
4. gnomAD v4. 955 000 exomes. 2021 (https://gnomad.broadinstitute.org/news/2023-11-gnomad-v4-0/).
5. Blended Genome Exome (BGE) Wave 1. 82 000 exomes. 2023. (Howrigan *et al.* 2022)
  The *All of Us* research project also used the VDS Combiner, but started from a group of Avro files each containing 4000 complete samples in a GVCF-like representation.
6. *All of Us*, April 2023 data freeze. 245 350 genomes. https://support.researchallofus.org/hc/en-us/article_attachments/17973653017236/_QC_Report_v7_release.pdf.

In addition to producing the VDS for each of these datasets, the Hail system was used to perform quality control and analyze each dataset.

The VDS Combiner is implemented in Hail Query, an open-source, partitioned, horizontally scalable, spot-tolerant, dataframe system and genomic analysis library with a Python API. Hail Query, and therefore the VDS Combiner, can run on any Apache Spark or Hail Batch cluster. A managed version of the former can be found in all the major clouds including AWS, Google, Azure, and Alibaba. The entire Hail software system is available at https://github.com/hail-is/hail. Documentation of how to use the VDS Combiner to produce a VDS is available in the Hail documentation at https://hail.is/docs/0.2/.

Unlike other joint calling systems, Hail Query is also a general purpose analysis system supporting relational algebra (e.g. filter, aggregate, group-by, order-by), distributed linear algebra (e.g. PCA, matrix multiplication), and export to many formats (e.g. BGEN, PLINK, VCF, TSV). The Hail Query language, which is used to manipulate values within datasets, supports a wide variety of data types (e.g. integers, floating-point numbers, strings, genomic loci, genotype calls, arrays, sets, dictionaries, tuples, ndarrays, and structs) and a wide variety of operations on these types (e.g. random functions, statistical distributions, statistical tests, linear and logistic regression, linear algebra, collection iteration, and aggregation).

### 3.2 Size in bytes

**Figure 5.**
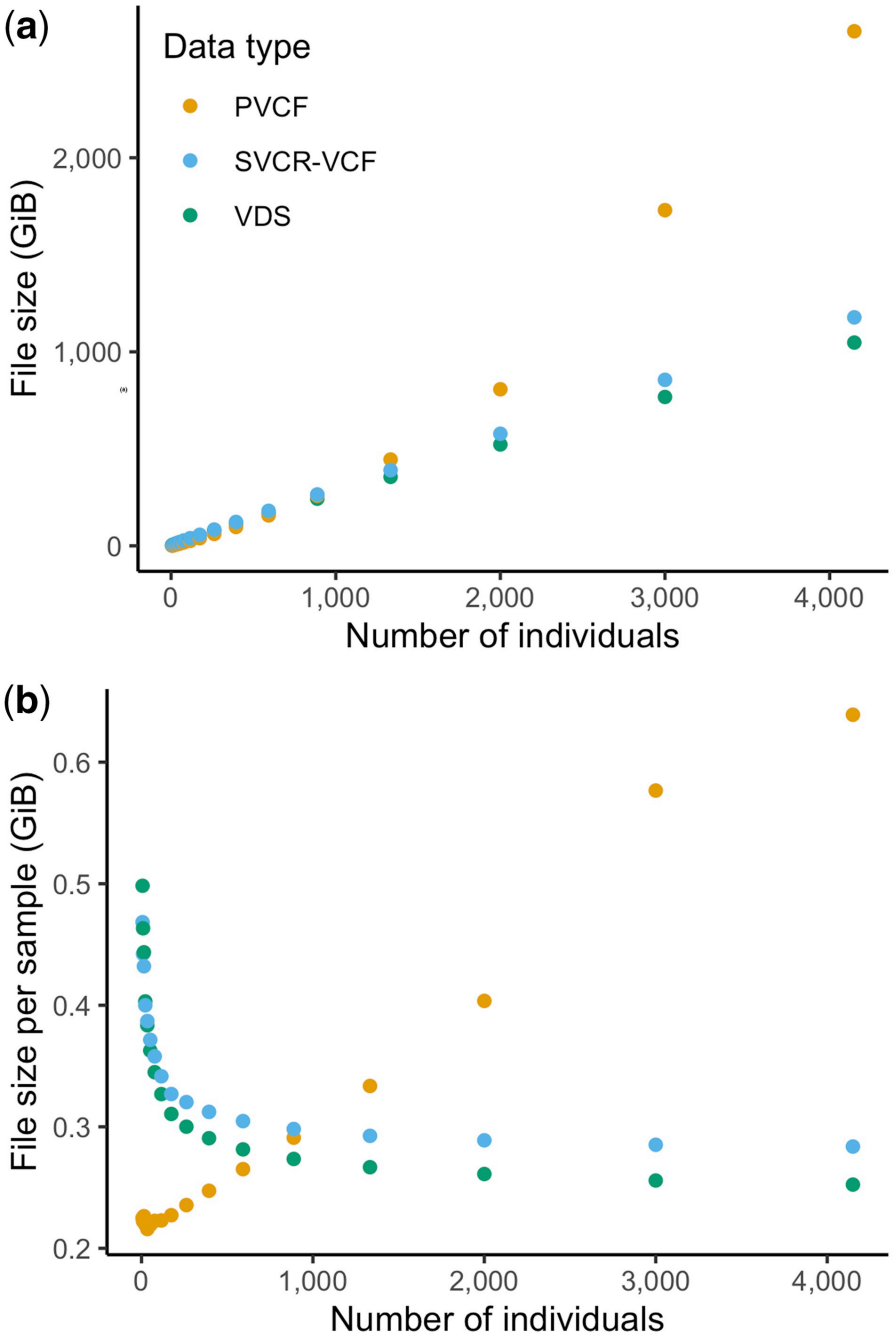
Scaling of dataset size of sequenced whole genomes represented in Hail LZ4-compressed VDS, gzip-compressed SVCR-VCF and gzip-compressed PVCF. In (a) total size in Gibibytes is plotted on the *y* axis, and in (b), plotting the size per sample (total size divided by number of samples) provides a clear view of the super-linear scaling of PVCF.

Consider subsets of the HGDP + 1kG dataset in PVCF, SVCR-VCF, and VDS. The total size grows super-linearly with sample size for PVCF but linearly for SVCR-VCF and VDS (Fig. 5a). Further confirming this, we see the size per sample increases for PVCF, while approaching an asymptote for SVCR-VCF and VDS (Fig. 5b), as expected since each human genome contains an approximately fixed amount of information.

It is critical to note that exact figures of size per sample should not be used to compare representations and formats without additional context that reflects the granularity and type of information contained. For example, choices in resolution of reference blocks and the selection of included FORMAT fields can lead to a substantial size difference in single-sample GVCFs for the same sequencing experiments, and these ratios are preserved in SVCR. Moreover, a necessary feature of any large-scale sequencing representation is a linear relationship between size and sample count, and we demonstrate that SVCR-VCF and VDS both scale linearly while PVCF scales super-linearly.

**Figure 6.**
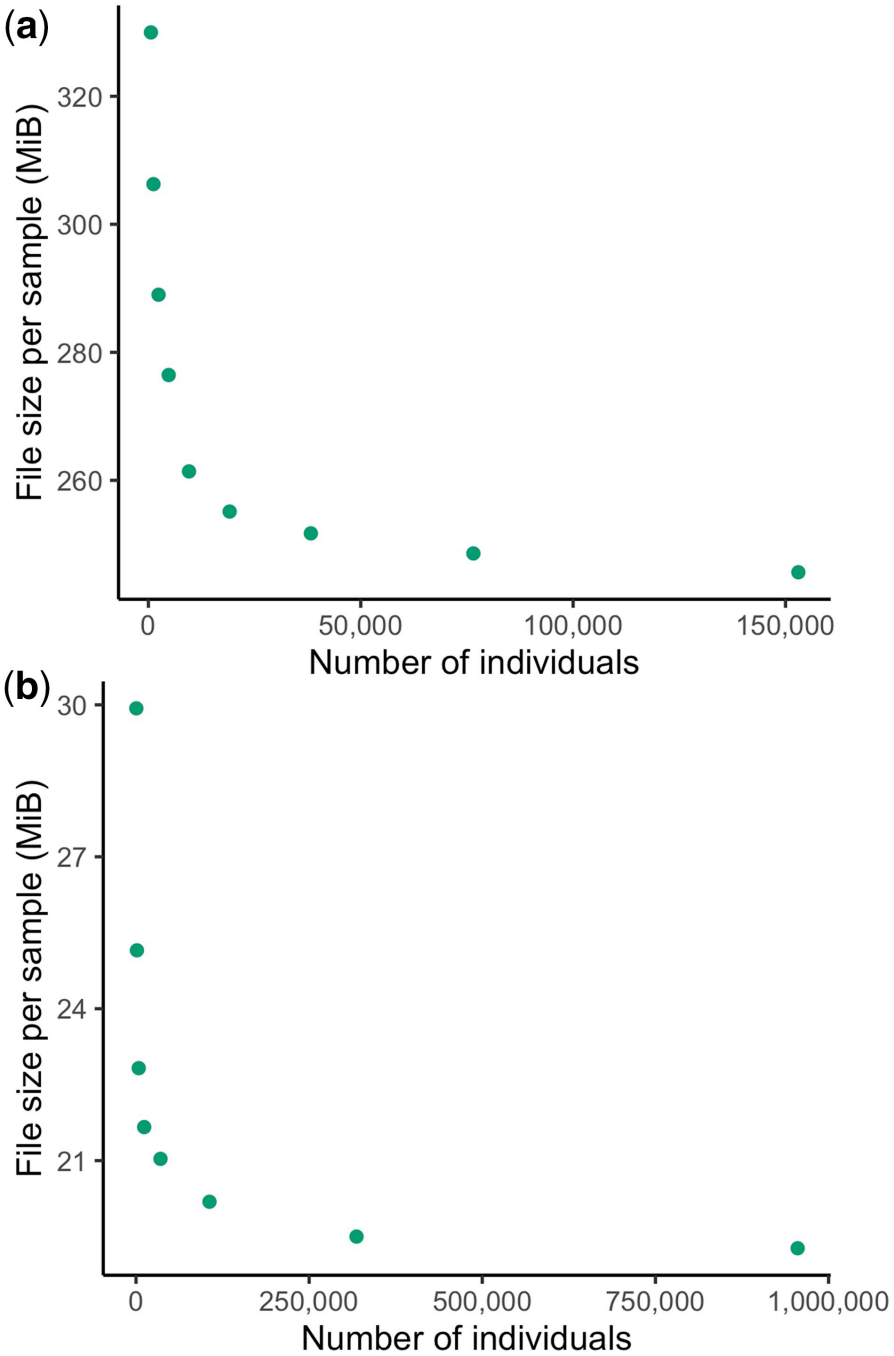
The growth of size in bytes per sample of subsets (a) gnomAD v3.1 exomes and (b) of gnomAD v4 exomes represented as VDS.

We did not systematically generate PVCFs or SVCR-VCFs for larger sample sizes due to the high cost of these experiments, but we show that this asymptotic trend persists in a VDS at larger cohort sizes with subsets of gnomAD (Fig. 6), and list a few selected examples of PVCF and VDS size in Table 3.

**Table 3.** Sizes and size per sample of selected large callsets.^a^

Data	Year	Number of Samples	Size (TiB)			Size per sample (MiB)		
			PVCF	GVCF	VDS	PVCF	GVCF	VDS
gnomAD v2 (G)	2016	15 496	16.00	5.00		1082	338.3	
gnomAD v3.1 (G)	2019	153 030			37.66			258.0
CCDG genomes	2021	136 959			108.7			832.4
*All of Us* April 2023 Data Freeze (G)	2023	245 350		292.6	20.95		1250	89.54
CCDG exomes	2021	203 664			3.36			17.30
gnomAD v4 (E)	2021	955 359			18.30			20.09
BGE Wave 1 (E)	2023	82 000		8.76	2.07		112.02	26.47

a In every case, PVCF is a bgzip-compressed Project VCF and GVCF is a bgzip-compressed VCF. A blank PVCF cell indicates that a PVCF was never generated. A blank GVCF cell indicates the sum-total size of the GVCFs is no longer available. The large variability in size per sample is due to variability in the granularity of reference blocks. In particular, integer precision GQs produce very large files. GVCF sizes should not be compared directly to other representations to assess compression, because in many cases some fields are dropped during or after combining GVCFs. G denotes genomes, E denotes exomes.

### 3.3 Cost of joint calling

As of August 2023, using Hail 0.2.120, a VDS produced from GVCFs costs approximately 0.005 USD per exome (amortized over 82 000 exomes), and in a large-scale application of the VDS combiner for whole genomes in 2019, we observed a cost of 0.10 USD per genome (amortized over 153 030 genomes). These numbers represent the sum total cost of using a Google Dataproc cluster with n1-standard spot instances to generate a VDS.

### 3.4 Cost of storage

The VDS is stored as a hierarchy of files or blobs. We observe from Fig. 5 that the cost per-sample of a VDS of at least 4000 samples is no more than 260 MiB per genome which is about 0.0052 USD per genome per month (at current costs of 0.02 USD per GiB per month). As previously discussed, the size of a VDS depends substantially on choices such as reference block granularity and sequencing fields included. In this case, we used GVCFs generated by GATK without reference block splitting, and retaining the usual genotype fields: *(L)GT*, *(L)AD*, *(L)PL*, *DP*, and *GQ*. In our experience, exome VDSes are approximately one-tenth the size of genome VDSes with the same number of samples.

### 3.5 Cost and speed of analysis

We have not extensively compared the speed (and thus cost) of analyzing a dataset stored in an SVCR format relative to the same dataset in a legacy format, such as bgzip-compressed-PVCF or BCF, nor in a modern competing format like msVCF. However, we make three claims:

1. For compressed-plaintext formats, the speed and cost of decompressing and then parsing text dominates the added cost of indirection through local allele indices.
2. For sufficiently large datasets, the nonlinearity of densely representing global-allele-indexed fields ensures that the bottleneck of any analysis will be the throughput of the storage system. For example, consider gnomAD v3.1, which has 150 000 samples: if size indeed grows as N^1.5^, then a PVCF would be ∼387 times larger than an SVCR-VCF. As an imperfect reference point, consider that the VDS is 37.66 TiB.

In our experience, *densification* (making homozygous reference calls explicit at every site covered by a reference block) is indeed slow and expensive. We are eager to watch the community explore how to implement downstream analyses directly against the sparse representation.

## 4 Conclusion

### 4.1 A concise, portable, and analyzable representation

We have presented the Scalable Variant Call Representation, a concise representation of genome sequencing datasets that enables quality control and analysis of nearly one million whole exome sequences. We reified SVCR in the VCF format as SVCR-VCF. We also presented the Hail VDS, a format generated and consumed by the open-source Hail Query library.

### 4.2 Joint calling

“Joint calling” has historically referred to combining samples into one dataset and also possibly adjusting genotypes given information from other samples. In this paper, we use the term “joint calling” to mirror conventional descriptions of these processes; however, “combining” may be more precise. The Hail VDS Combiner does not adjust genotypes nor is the SVCR format affected by adjustment, although notably, GATK-based “joint calling” pipelines such as GenotypeGVCFs do not do so either. Instead, SVCR and the VDS Combiner provide a representation and platform on which genotype adjustment and joint quality control via INFO annotations could be implemented, but the primary challenge at scale is the transposition of single-sample GVCF files into a variant-major layout.

### 4.3 Standardization

Local allele indexing and reference blocks in multi-sample VCFs were under discussion for a few years at the hts-specs repository under issue 434 (https://github.com/samtools/hts-specs/pull/434) and issue 435. The two proposals were merged into issue 758 in February of 2024, discussed extensively, and finally merged into the draft VCF 4.5 specification in April of 2024. The standard differs from SVCR-VCF in a couple subtle ways:

1. Instead of an LA field containing one entry per allele, the draft VCF 4.5 specification has an LAA field containing one entry per alternate allele. We prefer LA due to its simplicity and expect our underlying formats to efficiently store the repeated leading zero.
2. The draft VCF 4.5 specification omits LGT. In the experience of analysts using the VDS over the past several years, this led to confusion due to defaulting to a naive use of GT, rather than its local-allele-indexed kin. Consider, e.g. that removing an allele from a variant site does not require modifying locally indexed fields but does require modifying globally indexed ones. In general, we prefer the cognitive simplicity of exclusively using locally indexed fields.

The style of local allele indexes in draft VCF 4.5 is already supported by bcftools and is present in msVCF.

### 4.4 Future directions

Sparse representations of genotype matrices offer an efficient way to store large-scale sequenced cohorts, but many analyses still expect dense matrices as inputs. Developing new methods for querying and modeling genotype matrices that operate directly on sparse and/or compressed data will lead to substantial savings of compute time in addition to storage. Further, representing missing genotypes in VDS or a split SVCR format using a third table (in addition to reference and variant data) can support efficient analysis when reference quality information is not necessary by obviating the need to read the reference data; however, determining the best approach to do so in the context of dynamic filtering makes this challenging.

### 4.5 Related work

*GLNexus* is both a tool and a particular modification of the PVCF format (Lin *et al.* 2018). GLNexus combines GVCFs into PVCFs in three steps: (i) find a unified set of loci, (ii) “project” genotype-level fields from each GVCF into the PVCF, and (iii) adjust genotype calls. Additionally, GLNexus proposes a new allele unification algorithm which introduces a new PVCF variant type: <MONOALLELIC>. This algorithm and a new variant are designed to generate variants that are “completely nonoverlapping, with mutually exclusive alleles.”

GLNexus stores genotype records in a key-value store, and splits the reference genome into a disjoint set of 30 kilobase “bins.” Each genotype record is associated with a key pair of the bin containing this record and the sample identifier. They use an LSM-tree based key-value store which permits the addition of new samples in amortized linear time and space. Genomic intervals may also be retrieved in amortized linear time. Lin *et al.* (2018) and Yun *et al.* (2021) report the runtime and PVCF size for several applications of GLNexus to produce a PVCF from GVCFs, including an exome cohort of nearly 250 000 samples and a genome cohort of nearly 23 000 samples. We summarize their results in Table 4.

**Table 4.** GLNexus performance.^a^

Description	Reference	Samples	**Time** **(core-hours/sample)**	**Size** **(MB/sample)**
Chromosome 2 of an exome, unspecified GLNexus	Lin *et al.* (2018)	50 000	0.005	Not reported
Exome, standalone GLNexus	Lin *et al.* (2018)	16 521	0.058	Not reported
Exome, distributed GLNexus	Lin *et al.* (2018)	243 953	0.23 (“thread”-hours)	28
Genome, distributed GLNexus	Lin *et al.* (2018)	22 609	7	Not reported
Genome, unspecified GLNexus	Yun *et al.* (2021)	2504	Not reported	387

a We collect here the time to generate and size of PVCFs generated by GLNexus. The difference between “standalone” and “distributed” GLNexus is described in Section 4 “Availability” of Lin *et al.* (2018).

The innovations of GLNexus and SVCR address different PVCF challenges. SVCR, as defined above, requires one row per locus; however we believe GLNexus PVCFs are otherwise compatible with local allele indexing and preservation of reference blocks. The GLNexus database preserves reference information and effectively uses local allele indexing because it exactly preserves the GVCF records. The 30 kilobase reference bins resolve the challenge of point-queries for a dense vector in a manner similar to our “split at fixed periods” approach. The Hail VDS Combiner does not adjust genotype calls; however an algorithm similar to the GLNexus empirical frequency prior could be implemented in Hail Query.

*GATK’s GenotypeGVCFs* command produces a PVCF from either one or more GVCFs or a GenomicsDB workspace. The largest callset known to the authors to use GenotypeGVCFs directly on GVCFs is the 91 796 sample ExAC unfiltered callset (Lek *et al.* 2016). The largest callset to use GenomicsDB is the 164 332 individuals with exome sequence data and 20 314 individuals with genome sequence data from the unfiltered gnomAD v2 callset (Karczewski *et al.* 2020). GenotypeGVCFs suffers from both of the problems described above: for every variant site, every sample must have a genotype and for every allele, every genotype must have quality metrics.

*GenomicsDB* (https://www.intel.com/content/dam/www/public/us/en/documents/white-papers/genomics-storing-genome-data-paper.pdf) and *TileDB* (Papadopoulos *et al.* 2016) are projects with a shared origin which use similar techniques of sparse matrix representations to support better scaling. TileDB is an open-source multidimensional array storage manager written in C++ with support for large dense and sparse arrays. TileDB-VCF is an open-source C++ library with Python and command line interfaces which includes schemas and functionality for variant stores, including the ability to ingest new samples and query slices. TileDB-VCF stores GVCF variant and reference block records directly without PL expansion in a sparse array, indicating that TileDB-VCF disk footprints will scale linearly in the number of samples. GenomicsDB is a separately maintained open-source project originally developed on TileDB with the same data model described above, and tighter integration with GATK.

*The multi-sample VCF (msVCF) and DRAGEN gVCF genotyper* were developed by Illumina for generating and representing a jointly called dataset (https://www.illumina.com/science/genomics-research/articles/gVCF-Genotyper.html; https://support-docs.illumina.com/SW/dragen_v42/Content/SW/DRAGEN/gVCFGenotyper.htm). The DRAGEN gVCF genotyper accepts either GVCFs or “multi-sample GVCFs” as input and produces an msVCF as output. This process involves three steps:

1. DRAGEN aggregates batches of GVCFs into *census files* and *cohort files*. A census file stores variant metadata and per-sample reference blocks for a batch. A cohort file is a GVCF-like format containing all the samples of a batch.
2. DRAGEN aggregates all census files into a single global census file.
3. DRAGEN uses the global census file to generate, per cohort file, an msVCF.

The first step is parallelizable per batch of GVCFs. If desired, the second and third steps can be done in a tree-like fashion which allows both scaling of samples as well as incremental addition of samples. The DRAGEN gVCF genotyper is available both on-sequencer and in the cloud.

The msVCF uses a slight variant of local allele indexing. Instead of “LA” they use “LAA” and instead of requiring and including an entry for the reference allele, they always omit the reference allele. In contrast, this paper requires the first entry of *LA* to always be 0. The msVCF defines the LPL and LGT fields equivalently to this paper. Similarly to VDS, but unlike SVCR-VCF, DRAGEN stores the reference blocks in a separate file, the census file. We believe a split version of SVCF-VCF could serve the needs of DRAGEN while also being interoperable with other tools.

DRAGEN 4.0 has generated a 500 000 sample cohort. Illumina’s cloud-based DRAGEN platform, Illumina Connected Analytics, has been tested with cohorts of at least 100 000 samples. Illumina reports a cost of “0.3 iCredits per sample.” The cost of an iCredit depends on the volume of iCredits purchased, but is roughly 1 USD as of 2023-11-20. DRAGEN also supports a “compact GVCF” representation which omits statistics only necessary for *de novo* variant calling in pedigrees. This innovation is compatible with the SVCR and VDS Combiner.

*The Genomic Variant Store*, developed by the Broad Institute’s Data Sciences Platform (DSP), is a Google BigQuery based solution to GVCF aggregation and variant storage. The GVS representation is a variant of SVCR. It consists of three tables: *vet* (variants), *ref_ranges* (reference blocks), and *alt_allele* (variants). The vet and ref_ranges table are indexed by genomic position. The alt_allele table is indexed by sample. Much like GLNexus, it preserves GVCF records and thus effectively uses local allele indexing. GVS stores reference and variant data in distinct tables; indeed, it inspired the Hail VDS split representation. Storing the variant information twice, once row-major and once column-major appears unique to GVS and allows for rapid access to a single sample’s sequence. GVS can export to both PVCF and Hail VDS. As of October 2022, GVS can produce a 10 000 sample joint callset in less than half a day at a cost of 0.06 USD per genome (https://terra.bio/scaling-variant-discovery-to-a-million-genomes-with-the-genomic-variant-store/). GVS, using Hail Query and BigQuery together, produced the *All of Us* April 2023 freeze callset, a 245 000 whole genome VDS.

*BCFTools* supports local allele indexing, albeit using the *LAA* name and omitting the entry mapping the local reference allele to the global reference allele (https://samtools.github.io/bcftools/bcftools.html). This definition of *LAA* matches that of msVCF, described above, and the draft VCF 4.5 specification.

*Other VCF-like projects:* Multiple projects propose better encodings and compression of PVCF data. Savvy (LeFaive *et al.* 2021) is a storage layer and C++ query API for efficient storage and queries of variant data. Savvy’s data model has the same scaling characteristics as PVCF. Lossless textual encodings of PVCF files have also been proposed. Lin *et al.* proposed spVCF which achieves O(N^1.1^) scaling of bytes in samples (Lin *et al.* 2021). Eggertsson *et al.* propose popvcf which demonstrates further improvements over spVCF but they do not report a scaling factor (https://github.com/DecodeGenetics/popvcf). Unlike Savvy, spVCF, and popvcf, the size in bytes of SVCR and VDS both scale as O(N) in the samples. Moreover, the VDS is integrated with the scalable Hail Query dataframe and linear algebra system.

*Genotype-only projects:* There are many ways to compactly store a matrix of genotypes without any sequencing quality metadata (e.g. without *LAD* and *LPL*). For example, PGEN (Chang *et al.* 2015) and BGT (Li 2016) both can store each genotype in as few as two bits. GQT (Layer *et al.* 2016) stores genotypes in terms of four bitmasks (one for each biallelic diploid call plus missing) and then compresses the bitmasks. While these formats yield impressive constant factors on space and speed, particularly when combined with SIMD instructions, their inability to represent the *LAD* and *LPL* fields renders them unable to perform sequencing quality control. We conjecture that large callsets will be released once in an SVCR-like representation, allowing consumers to perform sequencing cohort quality control, and once in an already quality controlled state, converted to genotype-only representation for analysis.

## Data Availability

No new data were generated or analyzed in support of this research.

## References

[btae746-B1] Chang CC , Chow CC, Tellier LCAM et al Second-generation PLINK: rising to the challenge of larger and richer datasets. Gigascience 2015;4:7. 25722852 10.1186/s13742-015-0047-8 PMC4342193

[btae746-B2] Chen S , Francioli LC, Goodrich JK et al; Genome Aggregation Database Consortium. A genomic mutational constraint map using variation in 76,156 human genomes. Nature 2024;625:92–100. 10.1038/s41586-023-06045-0 38057664 PMC11629659

[btae746-B3] Danecek P , Auton A, Abecasis G et al; 1000 Genomes Project Analysis Group. The variant call format and VCFtools. Bioinformatics 2011;27:2156–8. 10.1093/bioinformatics/btr330 21653522 PMC3137218

[btae746-B5] Howrigan D , DeFelice M, Grimsby J et al Replacing GWAS arrays: capturing genomic diversity with a novel whole-exome plus low-pass whole genome product. Eur Neuropsychopharmacol 2022;63:e25–6.

[btae746-B6] Karczewski KJ , Francioli LC, Tiao G et al; Genome Aggregation Database Consortium. The mutational constraint spectrum quantified from variation in 141,456 humans. Nature 2020;581:434–43. 10.1038/s41586-020-2308-7 32461654 PMC7334197

[btae746-B7] Koenig ZAN , Yohannes MT, Nkambule LL et al A harmonized public resource of deeply sequenced diverse human genomes. *Genome Res* 2024;34:796–809. 10.1101/gr.278378.12338749656. 38749656 PMC11216312

[btae746-B8] Layer RM , Kindlon N, Karczewski KJ et al; Exome Aggregation Consortium. Efficient genotype compression and analysis of large genetic-variation data sets. Nat Methods 2016;13:63–5. 10.1038/nmeth.3654 26550772 PMC4697868

[btae746-B9] LeFaive J , Smith AV, Kang HM et al Sparse allele vectors and the savvy software suite. Bioinformatics 2021;37:4248–50. 10.1093/bioinformatics/btab378 33989384 PMC9502232

[btae746-B10] Lek M , Karczewski KJ, Minikel EV et al; Exome Aggregation Consortium. Analysis of protein-coding genetic variation in 60,706 humans. Nature 2016;536:285–91. 10.1038/nature19057 27535533 PMC5018207

[btae746-B11] Li H. BGT: efficient and flexible genotype query across many samples. Bioinformatics 2016;32:590–2. 26500154 10.1093/bioinformatics/btv613 PMC5963361

[btae746-B12] Lin MF , Rodeh O, Penn J et al GLnexus: joint variant calling for large cohort sequencing. bioRxiv, 10.1101/343970, 2018, preprint: not peer reviewed.

[btae746-B13] Lin MF , Bai X, Salerno WJ et al Sparse project VCF: efficient encoding of population genotype matrices. Bioinformatics 2021;36:5537–8. 10.1093/bioinformatics/btaa1004 33300997 PMC8016461

[btae746-B14] Lu W , Gauthier LD, Poterba T et al CHARR efficiently estimates contamination from DNA sequencing data. Am J Hum Genet 2023;110:2068–76. 10.1016/j.ajhg.2023.10.011 38000370 PMC10716339

[btae746-B15] Papadopoulos S , Datta K, Madden S et al The TileDB array data storage manager. Proc VLDB Endow 2016;10:349–60. 10.14778/3025111.3025117

[btae746-B17] Yun T , Li H, Chang PC et al Accurate, scalable cohort variant calls using DeepVariant and GLnexus. Bioinformatics 2021;36:5582–9. 10.1093/bioinformatics/btaa1081 33399819 PMC8023681

